# A nuclear lamina‐chromatin‐Ran GTPase axis modulates nuclear import and DNA damage signaling

**DOI:** 10.1111/acel.12851

**Published:** 2018-12-19

**Authors:** Natalia Dworak, Dawid Makosa, Mandovi Chatterjee, Kasey Jividen, Chun‐Song Yang, Chelsi Snow, William C. Simke, Isaac G. Johnson, Joshua B. Kelley, Bryce M. Paschal

**Affiliations:** ^1^ Center for Cell Signaling University of Virginia Charlottesville Virginia; ^2^ Department of Biochemistry and Molecular Genetics University of Virginia Charlottesville Virginia; ^3^ Department of Molecular and Biomedical Sciences University of Maine Orono Maine

## Abstract

The Ran GTPase regulates nuclear import and export by controlling the assembly state of transport complexes. This involves the direct action of RanGTP, which is generated in the nucleus by the chromatin‐associated nucleotide exchange factor, RCC1. Ran interactions with RCC1 contribute to formation of a nuclear:cytoplasmic (N:C) Ran protein gradient in interphase cells. In previous work, we showed that the Ran protein gradient is disrupted in fibroblasts from Hutchinson–Gilford progeria syndrome (HGPS) patients. The Ran gradient disruption in these cells is caused by nuclear membrane association of a mutant form of Lamin A, which induces a global reduction in heterochromatin marked with Histone H3K9me3 and Histone H3K27me3. Here, we have tested the hypothesis that heterochromatin controls the Ran gradient. Chemical inhibition and depletion of the histone methyltransferases (HMTs) G9a and GLP in normal human fibroblasts reduced heterochromatin levels and caused disruption of the Ran gradient, comparable to that observed previously in HGPS fibroblasts. HMT inhibition caused a defect in nuclear localization of TPR, a high molecular weight protein that, owing to its large size, displays a Ran‐dependent import defect in HGPS. We reasoned that pathways dependent on nuclear import of large proteins might be compromised in HGPS. We found that nuclear import of ATM requires the Ran gradient, and disruption of the Ran gradient in HGPS causes a defect in generating nuclear γ‐H2AX in response to ionizing radiation. Our data suggest a lamina–chromatin–Ran axis is important for nuclear transport regulation and contributes to the DNA damage response.

## 1 INTRODUCTION

The Ran GTPase plays a central role in regulating nuclear import and export in eukaryotic cells. By analogy with other GTPases, distinct conformations of Ran associated with its GTP‐ and GDP‐bound states are the basis for selective binding to the nuclear transport machinery (Pemberton & Paschal, 2005). Ran regulation of key steps in nuclear transport has been defined using biological, biochemical, and structural approaches (Chook et al., 1999; Pemberton & Paschal, 2005). Proteins that contain a nuclear localization signal (NLS) bind an NLS receptor (termed importin‐α or KPNA) and assemble into a cytoplasmic NLS‐KPNA‐importin‐β complex that translocates through the nuclear pore complex (NPC). Upon reaching the nucleoplasm, RanGTP binding to a single, high‐affinity site on importin‐β triggers disassembly of the NLS‐KPNA‐importin‐β complex (Görlich, Panté, Kutay, Aebi, & Bischoff, 1996), thus releasing the NLS‐containing proteins for nuclear function. RanGTP, therefore, regulates nuclear import by controlling complex disassembly, the terminal step in this pathway. By contrast, RanGTP regulates the initial step of nuclear export by promoting export complex assembly. The major pathway for transporting nuclear export signal (NES)‐containing proteins from the nucleus to the cytoplasm is mediated by the NES receptor Crm1. The NES‐Crm1‐RanGTP complex forms in the nucleoplasm, translocates through the NPC, and is disassembled in the cytoplasm because the complex encounters the GTPase‐activating protein (GAP) for Ran (Askjaer et al., 1999; Bischoff, Klebe, Kretschmer, Wittinghofer, & Ponstingl, 1994). GAP stimulation of GTP hydrolysis promotes disassembly of NES‐Crm1‐RanGTP complex, which releases the NES‐containing protein for function in the cytoplasm.

The aforementioned design of nuclear transport, which is conserved from yeast to human, creates an ongoing demand for RanGTP production in the nucleus. This includes the need to replenish nuclear Ran protein, which continuously exits the nucleus as a component of export complexes. Cytoplasmic RanGDP is recognized by the nuclear import factor NTF2; the RanGDP‐NTF2 complex (Paschal, Delphin, & Gerace, 1996; Ribbeck, 1998; Smith, Brownawell, & Macara, 1998) translocates through the NPC where it encounters the Ran guanine nucleotide exchange factor (GEF), regulator of chromatin condensation 1 (RCC1) (Ohtsubo, Okazaki, & Nishimoto, 1989). RCC1 promotes nucleotide exchange of RanGDP to RanGTP (Bischoff & Ponstingl, 1991). NTF2 and RCC1, therefore, provide the essential functions of (a) maintaining the Ran protein levels and (b) regenerating RanGTP levels in the nucleus that are crucial for nucleocytoplasmic transport pathways. An important feature of Ran regulation is the mutually exclusive cellular distribution of RanGAP and RCC1. RanGAP is a cytoplasmic enzyme and includes a pool anchored to the outer surface of the NPC (Mahajan, Gerace, & Melchior, 1998; Matunis, Wu, & Blobel, 1998) where it encounters export complexes. RCC1 is restricted to the nucleus, where it binds chromatin and undergoes a cycle of chromatin binding and dissociation as part of nucleotide exchange (Nemergut, 2001; Ohtsubo et al., 1989). The cellular distribution of RanGAP and RCC1 generates compartment identity by restricting RanGTP production to the nucleus, and ensuring that RanGTP that leaves the nucleus as an export complex is efficiently converted to RanGDP.

Under steady‐state conditions, Ran is concentrated in the nucleus, and by immunofluorescence (IF) microscopy, it displays a nuclear:cytoplasmic ratio of ~3:1 which is termed the Ran protein gradient. Our laboratory found that the Ran protein gradient is disrupted in primary fibroblasts from patients with Hutchinson–Gilford progeria syndrome (HGPS) (Kelley et al., 2011). HGPS is caused by a sporadic mutation in exon 11 of the LMNA gene (Eriksson et al., 2003) that results in a preLamin A protein processing defect. The exon 11 mutation occurs within a cryptic splicing site; utilization of this site by the splicing machinery generates an mRNA with an internal 150‐nucleotide deletion (Eriksson et al., 2003). Subsequent translation produces a mutant Lamin A protein that lacks the endoproteolytic cleavage site for the nuclear membrane‐associated enzyme Zmpste24. PreLamin A cleavage normally occurs after Lamin A undergoes farnesylation and nuclear membrane attachment (Rusinol & Sinensky, 2006). In HGPS cells, the progerin form of Lamin A is uncleaved and is therefore constitutively anchored to the inner nuclear membrane. Progerin exerts dominant negative effects on nuclear morphology and causes a reduction in the heterochromatin marks Histone H3K9me3 and Histone H3K27me3 (Scaffidi & Misteli, 2005, 2006 ; Shumaker et al., 2006). The Ran gradient defect in HGPS fibroblasts can be rescued by inhibiting farnesylation of progerin, a treatment that also restores heterochromatin (Kelley et al., 2011). These and other findings led us to propose that alterations in the nuclear lamina are transmitted to chromatin, which, in turn, is relayed to the Ran GTPase system. A strong candidate protein that could sense and transmit chromatin changes to Ran is RCC1. The premise for this view includes the chromatin association of RCC1, data showing that the RanGDP‐RCC1 nucleotide exchange reaction uses chromatin as a scaffold (Nemergut, 2001), and the fact that RCC1‐chromatin dynamics measured by FRAP are altered by progerin expression (Kelley et al., 2011). Moreover, reducing the nuclear level of functional RCC1 protein via a temperature‐sensitive allele (Tachibana et al., 2000; Tachibana, Imamoto, Seino, Nishimoto, & Yoneda, 1994) disrupts the Ran gradient to an extent comparable to the Ran disruption observed in HGPS patient cells (Kelley et al., 2011). Thus, RCC1 is a chromatin‐binding protein that is required to form and maintain the Ran gradient.

Here, we set out to examine whether heterochromatin can regulate the Ran GTPase systems, which we tested by reducing the activity of G9a and G9a‐like protein (GLP), major histone methyltransferases (HMTs) that form heteromeric complexes in mammalian cells. We show that HMT depletion, and application of HMT inhibitors, reduces heterochromatin levels and disrupts the Ran protein gradient in normal human fibroblasts. Reducing heterochromatin levels in budding yeast by deletion of the HMT Set2 also disrupted the Ran distribution. Based on chromatin immunoprecipitation (ChIP) data indicating that RCC1 can be enriched on chromatin marked with H3K9me3 and H3K27me3, we propose that RCC1 senses heterochromatin levels, which, in turn, is transduced to Ran. Decrease in heterochromatin levels and disruption of the Ran gradient with the HMT inhibitor Bix01294 were sufficient to induce a defect in nuclear import of the “large cargo” protein TPR, an effect elicited by progerin (Snow, Dar, Dutta, Kehlenbach, & Paschal, 2013). This led us to explore whether DNA damage signaling, a pathway that relies on nuclear import of large proteins cargoes, is affected by the state of the nuclear lamina, heterochromatin, and Ran. In cells where the Ran gradient is disrupted, we observed a striking reduction in nuclear localization of ATM and H2AX protein and reduced generation of nuclear γ‐H2AX in response to ionizing radiation. Our data suggest that chromatin regulation of Ran is a conserved mechanism and that the lamina–chromatin–Ran axis in mammalian cells promotes the DNA damage response through nuclear transport‐based mechanisms.

## 2 RESULTS

**Figure 1:**
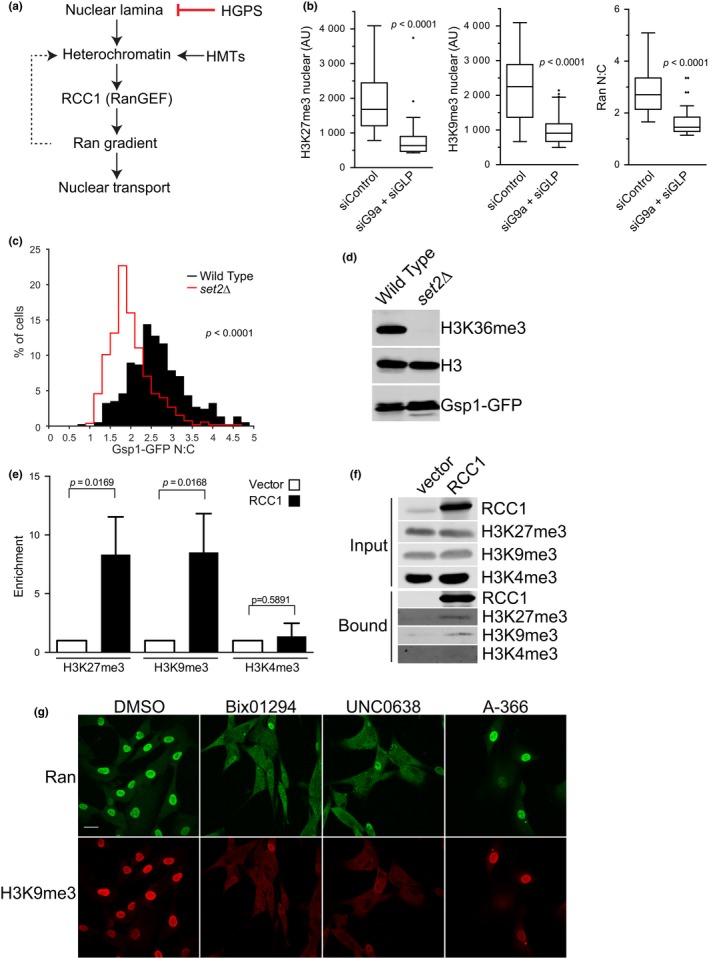
Chromatin regulation of the interphase Ran gradient. (a) Working model for a nuclear lamina–chromatin–Ran axis. The dotted line reflects Ran gradient‐dependent formation of heterochromatin. (b) Depletion of HMTs G9a and GLP reduces heterochromatin marks and disrupts the Ran gradient. siRNAs were transfected into normal human fibroblasts (4 days), which were subsequently stained for chromatin marks and endogenous Ran, imaged by wide‐field IF microscopy, and quantified. Representative images from this analysis, and confirmation that the siRNAs reduced G9a and GLP message levels, are also provided (Figure S1a–c). (c) Deletion of the HMT Set2 disrupts the Ran gradient in S. cerevisiae. WT and *set2* deletion strains containing GFP‐tagged GSP (*S. cerevisiae* Ran) were analyzed by quantitative fluorescence microscopy. (d) Immunoblotting showing loss of the heterochromatin mark H3K36me3 in the set2 deletion strain. (e) Quantification of heterochromatin enrichment by ChIP for RCC1. Bar graphs depict the mean values for enrichment (±*SD*) from three experiments. (f) ChIP and immunoblotting for RCC1 and chromatin marks. The samples were eluted with gel sample buffer, and input (0.5%) bound fractions (12.5%) were immunoblotted for RCC1 and modified forms of Histone H3. The sensitivity of the H3K9me3 and H3K27me3 antibodies was established by serial dilution of extracts (Figure S1e). (g) Effects of histone methyltransferase G9a inhibitors on the Ran gradient and H3K9me3 levels in human fibroblasts. Chemical inhibitors (Bix01294, UNC0638, A‐366) were applied to human fibroblasts and endogenous Ran and Histone H3K9me3 levels examined by IF microscopy. Scale bar 20 µm

In primary fibroblasts from Progeria patients, alterations in the structure of the nuclear lamina are associated with reduced levels of the heterochromatin marks Histone H3K9me3 and Histone H3K27me3, as well as lower nuclear levels of HP1 (Kelley et al., 2011; Scaffidi & Misteli, 2006). Our group showed that the reduction in heterochromatin in Progeria was correlated with changes in the nuclear:cytoplasmic (N:C) levels of the Ran GTPase, the master regulator of nuclear transport (Datta, Snow, & Paschal, 2014; Kelley et al., 2011). This led us to suggest that heterochromatin might regulate the Ran GTPase gradient through RCC1, a chromatin‐binding protein that mediates nucleotide exchange on Ran (Ohtsubo et al., 1989). Consistent with this possibility, genomewide localization of the RCC1 homologue in yeast, Prp20, showed preferential binding to inactive genes (Casolari et al., 2004). From biochemical analysis, it is known that RCC1 binds histones and DNA, and the nucleotide exchange reaction that converts RanGDP to RanGTP involves transient formation of a Ran:RCC1:chromatin complex (Hao & Macara, 2008; Nemergut, 2001). Histones can stimulate RCC1‐mediated nucleotide exchange on Ran, and the crystal structure of RCC1 bound to the nucleosome revealed the specific contacts between RCC1, histones, and DNA (Makde, England, Yennawar, & Tan, 2010; Nemergut, 2001). These data lend strong support for the model that RCC1 uses chromatin as a scaffold for the nucleotide exchange reaction. If this reaction requires or is biased toward heterochromatin, then conditions that reduce heterochromatin levels in the nucleus could affect Ran:RCC1:chromatin formation, generation of RanGTP, and Ran regulation of nuclear transport pathways. These relationships can be viewed as an axis that links the structure of the nuclear lamina and chromatin state to the activity of the nuclear transport machinery (Figure 1a).

To explore the relationships in the axis, we first tested whether reducing the expression of HMTs linked to heterochromatin formation had an impact on Ran distribution. As expected, siRNA depletion of the HMTs G9a and GLP, which have fundamental roles in heterochromatin formation by acting as mono‐ and dimethylases, reduced the nuclear levels of H3K27me3 and H3K9me3 in normal human fibroblasts detected by immunofluorescence (IF) microscopy (Figure 1b; Figure S1a–c). Depletion of the HMTs reduced the N:C distribution of endogenous Ran, also detected by IF microscopy. These data provide evidence that chromatin can act upstream of Ran distribution in mammalian cells.

The core components and major regulatory features of the Ran GTPase system are conserved in diverse species including *S. cerevisiae*. The Ran homologue, Gsp1, is imported into the nucleus by the essential transport factor NTF2 (Corbett & Silver, 1996; Paschal, Fritze, Guan, & Gerace, 1997) where it becomes concentrated relative to the cytoplasm (Belhumeur et al., 1993). GDP‐GTP exchange is catalyzed by Prp20, which is homologous to, and complemented by, human RCC1 (Clark, Ohtsubo, Nishimoto, Goebl, & Sprague, 1991; Fleischmann et al., 1991). To test whether chromatin structure can modulate the Gsp1 gradient in *S. cerevisiae*, we tagged endogenous Gsp1 with GFP and measured the N:C ratios in a WT strain and a strain deleted for *set2*, the methyltransferase responsible for generating H3K36me3 as a part of silencing mechanisms (Suzuki et al., 2016). In WT cells, Gsp1 forms an N:C gradient of ~3:1; however, the ratio is reduced to ~2:1 with loss of H3K36me3 (Figure 1c,d; Figure S1d). Thus, the epigenetic state of chromatin can influence the interphase Ran distribution in a simple eukaryote as well.

We next tested whether RCC1 can bind heterochromatin in mammalian cells by chromatin immunoprecipitation (ChIP). We transfected 293 T cells with Flag‐RCC1 and used anti‐Flag antibody to prepare RCC1‐chromatin samples. After reversal of the crosslinks, the samples were analyzed by immunoblotting with antibodies specific for Histone H3 marked with H3K4me3, H3K9me3, and H3K27me3. We observed statistically significant RCC1‐dependent recovery of heterochromatin marked by H3K9me3 (*p* = 0.0169) and H3K27me3 (*p* = 0.0168), but minimal binding of open chromatin marked by H3K4me3 (Figure 1e,f; data pooled from three experiments). The quantity of H3K9me3 and H3K27me3 recovered represented 1.14% and 0.69%, respectively, of the input. The H3K9me3 and H3K27me3 antibodies are capable of detecting the respective modifications over a broad range of sample input (Figure S1e). These data, together with studies showing that RCC1 exchange activity is stimulated by histones (Nemergut, 2001) and the RCC1‐nucleosome co‐crystal (Makde et al., 2010), support the model whereby RCC1 uses heterochromatin as a scaffold in the context of generating a Ran gradient in interphase cells.

As another approach for assessing whether heterochromatin is important for the interphase Ran gradient, we employed chemical inhibitors to G9a and GLP. HMT inhibitors Bix01294 (Kubicek et al., 2007), UNC0638 (Vedadi et al., 2011), and A‐366 (Sweis et al., 2014) were each applied to human fibroblasts, and the distribution of endogenous Ran and Histone H3K9me3 was examined by IF microscopy. We found that the nuclear levels of Ran were reduced in response to each of the three HMT G9a/GLP inhibitors, though the effect of A‐366 appeared less penetrant than Bix01294 and UNC0638 (Figure 1g). The reduction in Histone H3K9me3 with Bix01294 occurs because trimethylation of this site by Suv‐39 h1 depends on dimethylation by G9a and GLP (Shinkai & Tachibana, 2011), as well as other levels of crosstalk between methyltransferases (Fritsch et al., 2010). By double‐label IF microscopy, cells that displayed a drug‐induced disruption in nuclear Ran localization had a corresponding reduction in Histone H3K9me3 (Figure 1g). Notably, the chemical inhibitor and siRNA effects on heterochromatin and Ran in normal fibroblasts are similar in magnitude to the changes caused by progerin expression in HGPS cells, which reduces heterochromatin levels and disrupts the Ran gradient in a manner that requires farnesylation of progerin (Kelley et al., 2011; Snow et al., 2013). We conclude from these experiments that heterochromatin functions upstream of the Ran gradient and that the chromatin effect could be transduced through RCC1, which is the only nucleotide exchange factor for Ran.

**Figure 2:**
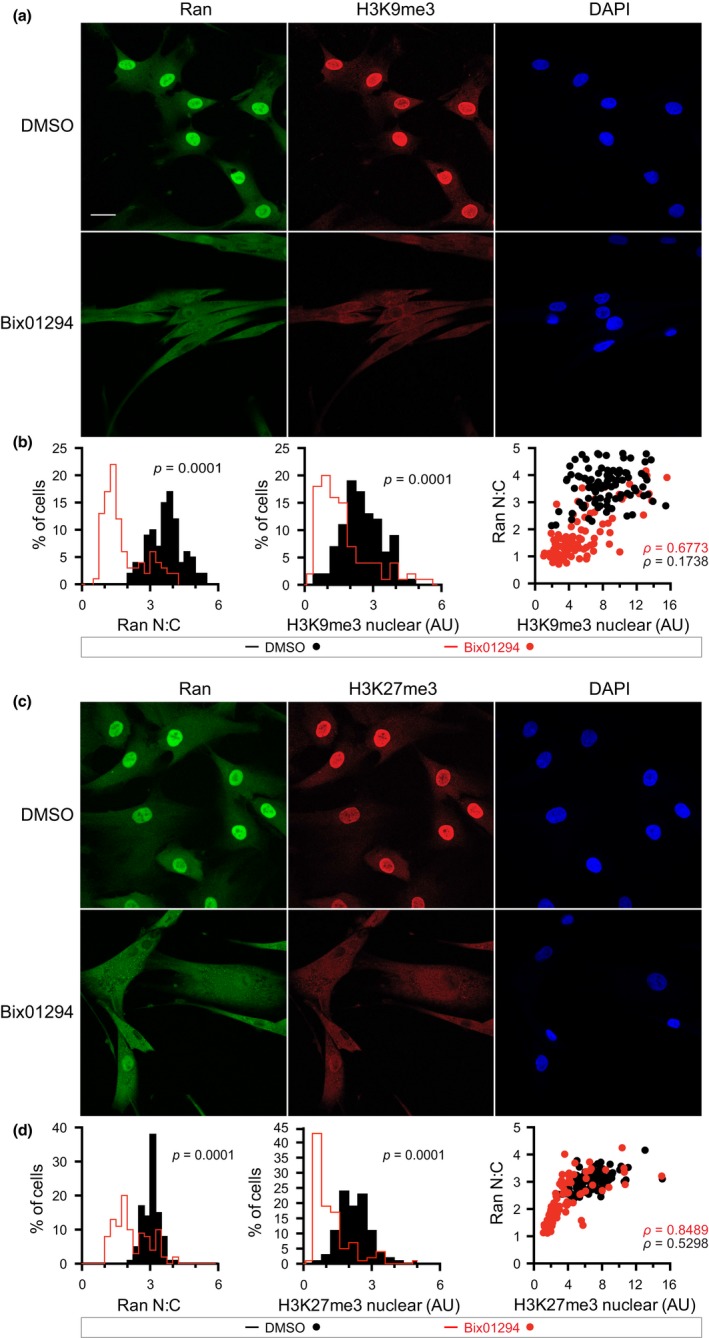
The G9a inhibitor Bix01294 reduces H3K9me3 and H3K27me3 and disrupts the interphase Ran gradient. (a) IF microscopy images of Ran and Histone H3K9me3 in human fibroblasts treated with DMSO and Bix01294. Scale bar 20 µm. (b) Histograms of Ran N:C and nuclear H3K9me3, and Ran N:C as a function of H3K9me3. (c) IF microscopy images of Ran and Histone H3K27me3 in human fibroblasts treated with DMSO and Bix01294. (d) Histograms of Ran N:C and nuclear H3K27me3, and Ran N:C as a function of H3K27me3

**Figure 3:**
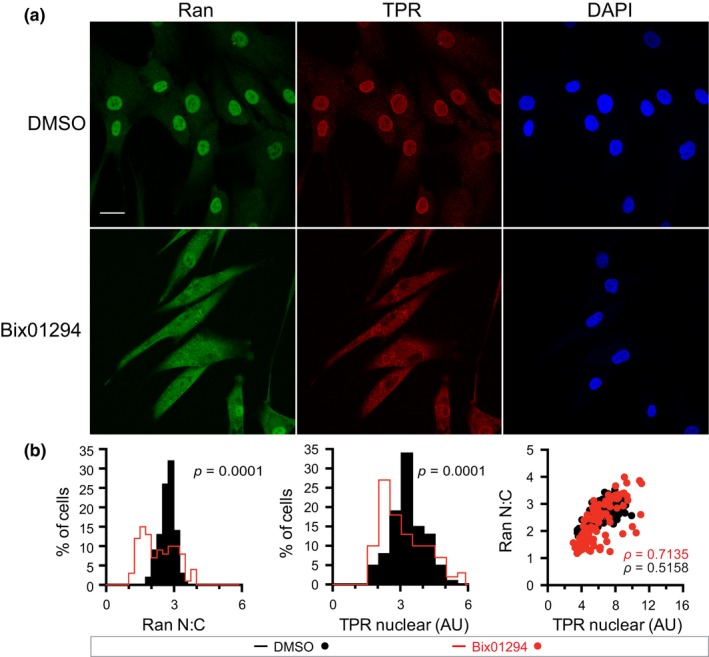
Bix01294 treatment inhibits nuclear import of TPR. (a) IF microscopy images of Ran and the nucleoporin TPR in human fibroblasts treated with DMSO and Bix01294. Scale bar 20 µm. (b) Histograms of Ran N:C and nuclear TPR, and Ran N:C as a function of nuclear TPR

We used quantitative IF microscopy to analyze the effects of Bix01294 with the goal of testing whether there is a correlation between heterochromatin levels and Ran distribution. Bix01294 treatment of human fibroblasts caused a significant reduction in Histone H3K9me3, H3K27me3, and the Ran N:C, resulting in a leftward shift of the histograms (Figure 2b,d). Moreover, the Ran N:C was correlated with H3K9me3 and H3K27me3, though the correlation appeared more striking when heterochromatin levels were reduced by Bix01294 treatment (Figure 2a–d). Disruption of the Ran gradient in Progeria has a strong inhibitory effect on nuclear import of the nucleoporin TPR, which provides a facile readout for the efficiency of Ran‐dependent transport on an endogenous protein (Kelley et al., 2011; Snow et al., 2013). Bix01294‐treated cells showed a reduction in TPR import, which by IF microscopy was visible as cytoplasmic TPR staining and reduced nuclear TPR signal (Figure 3a,b). From these results, we conclude that manipulating heterochromatin levels by a pharmacologic approach can disrupt the Ran gradient and reduce the nuclear import of an endogenous cargo in normal human fibroblasts.

**Figure 4:**
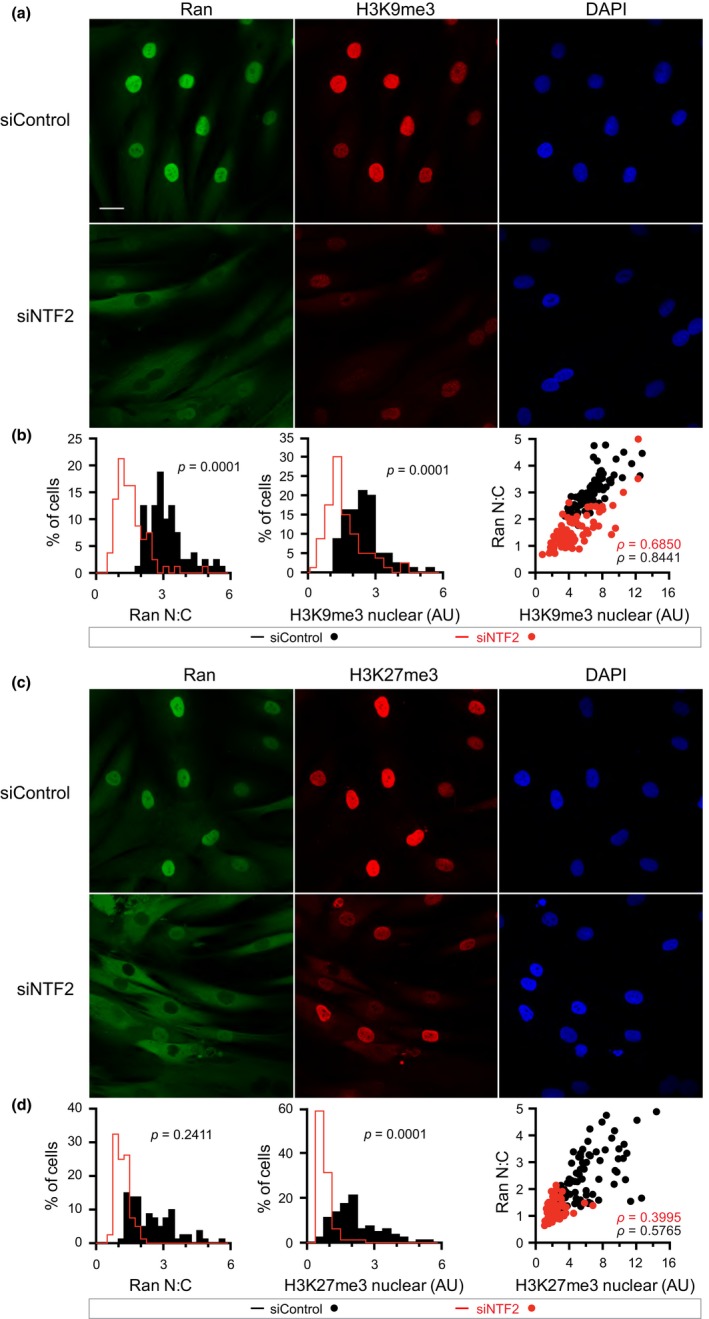
Depletion of the Ran import receptor NTF2 disrupts the Ran gradient and reduces heterochromatin levels. (a) IF microscopy images of Ran and H3K9me3 in human fibroblasts depleted of NTF2. Scale bar 20 µm. (b) Histograms of Ran N:C and nuclear H3K9me3, and Ran N:C plotted as a function of H3K9me3. (c) IF microscopy images of Ran and H3K27me3 in human fibroblasts depleted of NTF2. (d) Histograms of Ran N:C and nuclear H3K27me3, and Ran N:C plotted as a function of H3K27me3

One surprising effect of disrupting the Ran gradient in interphase cells is the selective inhibition of large cargo import (Snow et al., 2013). While the specific mechanism that underlies this selectivity is incompletely understood, the available data suggest that a lower level of RanGTP produced in the nucleus under conditions of Ran gradient disruption is sufficient to support nuclear import of small and intermediate‐sized proteins, but not large proteins (Snow et al., 2013). Since HMTs assemble into large, multi‐subunit complexes, we tested whether disruption of the Ran gradient affected heterochromatin levels. As shown previously (Datta et al., 2014), depletion of the Ran import factor NTF2 is sufficient to disrupt the Ran gradient (Figure 4). Ran gradient disruption by NTF2 depletion reduced the nuclear levels of Histone H3K9me3 and Histone H3K27me3 (Figure 4). Moreover, there is a correlation between Ran and the chromatin marks at both high (Spearman *p* = 0.001) and low (Spearman *p* = 0.001) Ran N:C values, indicating that the Ran gradient is limiting for the establishment and/or maintenance of these marks.

Progerin expression reduces Histone H3K9me3 and Histone H3K27me3 levels throughout the nucleus (Scaffidi & Misteli, 2005, 2006 ; Shumaker et al., 2006), showing its effects are not restricted to chromatin proximal to the nuclear envelope. Given the global effect of progerin on the nucleus, we considered whether other chromatin‐based pathways might be affected by changes in nuclear lamina structure. For example, cells from HGPS patients, and aged vascular smooth muscle cells expressing preLamin A, have been reported to accumulate unrepaired DNA (Liu, Rusinol, Sinensky, Wang, & Zou, 2006; Richards, Muter, Ritchie, Lattanzi, & Hutchison, 2011; Warren & Shanahan, 2011). Consistent with this notion, preLamin A expression was shown to reduce import of the DNA repair factor 53BP1 (Cobb et al., 2016). To determine whether there is an interplay between the nuclear lamina and DNA damage and repair pathways in normal fibroblasts, we made use of the HIV protease inhibitor lopinavir (LPV), which has the off‐target effect of inhibiting the preLamin A protease Zmpste24 (Coffinier et al., 2007). LPV treatment results in the accumulation of unprocessed, nuclear membrane‐tethered preLamin A (Caron et al., 2007). We have shown in previous work that LPV treatment is sufficient to disrupt the Ran gradient because preLamin A accumulation induces progerin‐like effects and that the LPV effect on Ran is lost with lamin A knockdown (Datta et al., 2014). An advantage of LPV is that it can be used on low‐passage normal cells where its effects are mediated through the accumulation of endogenous preLamin A. The LPV approach avoids potential artifacts caused by ectopic lamin A overexpression, effects associated with cell passage number that may, or may not, be linked to lamina structure, and the inherent phenotypic heterogeneity of cells from HGPS patients.

**Figure 5:**
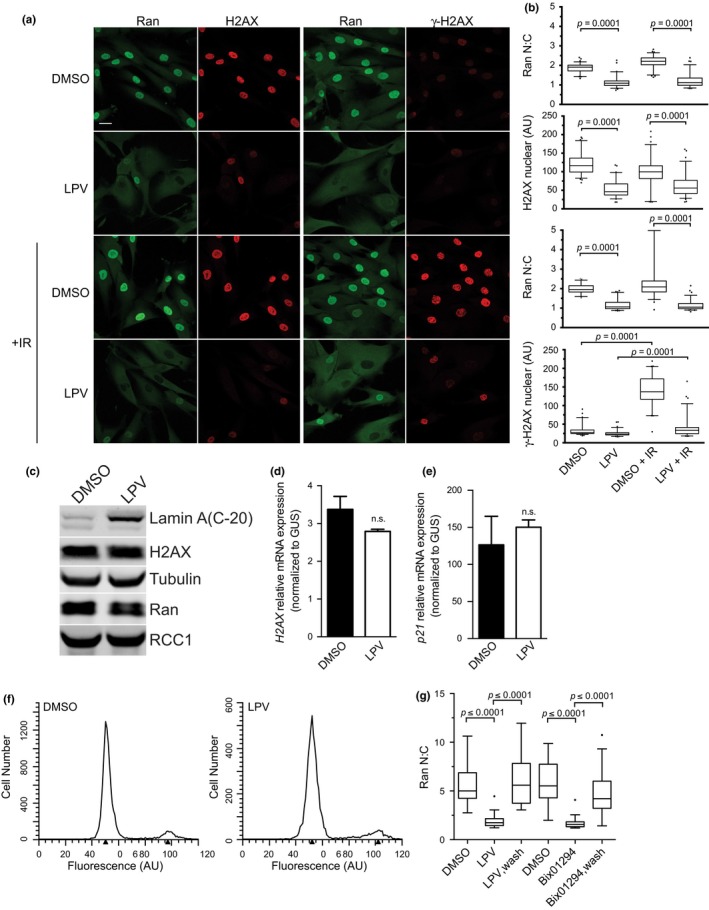
Inhibition of lamin A processing by LPV treatment disrupts the Ran gradient and reduces the nuclear DNA damage response. (a) IF microscopy images of Ran, H2AX, and γ‐H2AX in human fibroblasts exposed to IR in the presence of DMSO and LPV. (b) Quantitative analysis of Ran N:C and nuclear H2AX and γ‐H2AX. (c) Immunoblotting of human fibroblasts after treatment with LPV. (d) Expression of H2AX after treatment with LPV determined by RT–PCR. (e) Expression of p21 after treatment with LPV determined by RT–PCR. (f) Cell cycle profiles of human fibroblasts treated with LPV measured by propidium iodide (PI) staining. (g) Restoration of the Ran gradient after washout of LPV

We treated normal human fibroblasts with LPV for 72 hr, subjected the cells to IR (5 Gy), and stained for endogenous Ran and γ‐H2AX. Consistent with our previous data, LPV treatment disrupted the Ran gradient, visible as a decrease in nuclear staining and a slight increase in cytoplasmic signal (Figure 5a,b). Moreover, there was a striking defect in the ability of LPV‐treated cells to generate γ‐H2AX in response to IR (Figure 5a,b). These data can be explained, at least in part, by the reduced level of nuclear H2AX revealed by IF microscopy (Figure 5a,b). Some cells in LPV‐treated cultures retain a Ran gradient (Figure S2), and the same cells stain positive for γ‐H2AX after IR (Figure 5a,b). This might reflect LPV resistance or other phenotypic differences. LPV has no significant effect on H2AX message level measured by RT–PCR or H2AX protein level detected by immunoblotting, nor does LPV significantly affect the cell cycle profile examined by flow cytometry (Figure 5c,d,f). Thus, the reduced H2AX signal by IF could reflect a reduced level of H2AX import. Under these conditions, there is not a corresponding increase in cytoplasmic signal for H2AX. This may reflect the fact H2AX concentration would be at least 10‐fold lower in the cytoplasm compared to the nucleus because of the volumes of these compartments, but other explanations for the reduced nuclear level of H2AX are clearly possible. Our data indicate that membrane tethering of endogenous preLamin A and the ensuing disruption of the Ran gradient impair the generation of γ‐H2AX during the DNA damage response. LPV treatment under these conditions does not appear to induce senescence since there was not a significant change in p21 induction, and the LPV effects are reversed by washout of the drug (Figure 5e,g).

We explored whether the aforementioned effects of LPV on γ‐H2AX induction are pertinent to signaling in Progeria patient cells, which display Ran gradient defects due to progerin expression (Kelley et al., 2011). Using fibroblasts from three different HGPS patients, we tested for IR induction of γ‐H2AX. We used cells at a relatively low passage number (<15) to minimize the secondary phenotypes such as membrane blebbing that can occur with high passage number, which might confound the results. By IF microscopy, HGPS 1498, HGPS 3199, and HGPS 1972 cells showed a clear deficit in generating γ‐H2AX in response to IR, though a few cells with nuclear γ‐H2AX were observed (Figure S3a). By immunoblotting, IR induction of γ‐H2AX was similar in WT and HGPS cells (Figure S3b). The strong reduction in nuclear g‐H2AX may, therefore, be a consequence of reduced import since Progeria cells showed lower levels of nuclear H2AX as well (Figure S3a). Thus, these three HGPS lines display a defect in γ‐H2AX generation in response to acute DNA damage. Our findings are consistent with data from another group using the HGPS patient line HGADFN167, which displayed a reduced ability to generate γ‐H2AX in response to the DNA‐damaging drugs, such as doxorubicin and camptothecin (Zhang et al., 2016).

**Figure 6:**
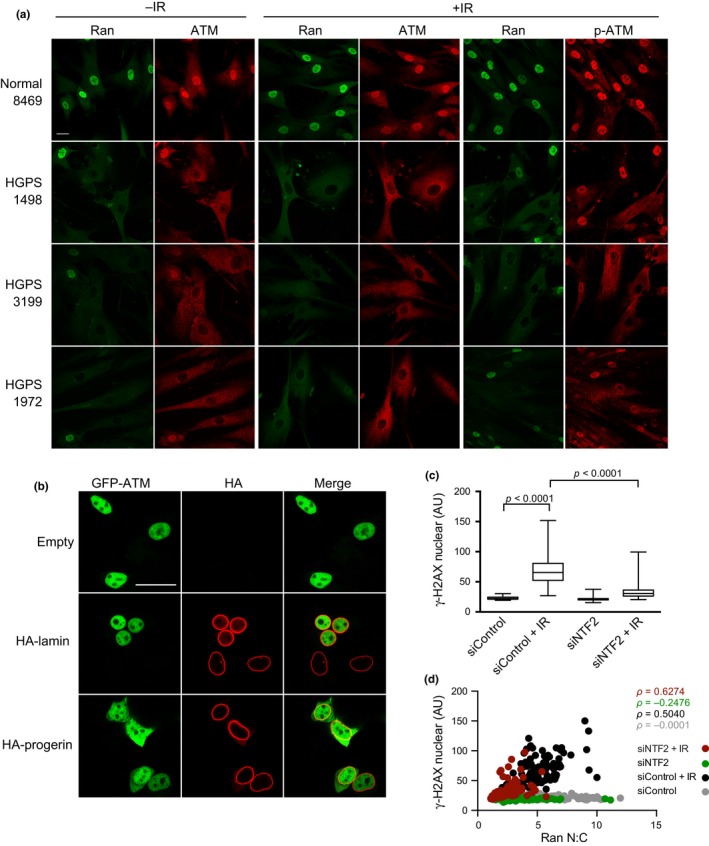
Disruption of the nuclear lamina in HGPS fibroblasts reduces ATM import and γ‐H2AX formation induced by IR. (a) IF microscopy images of Ran, ATM, and p‐ATM in HGPS fibroblasts in response to IR treatment. (b) Subcellular distribution of GFP‐ATM in cells co‐transfected with WT lamin A and progerin. Scale bar 20 µm. (c) Quantification of γ‐H2AX levels in cells depleted of the Ran import factor NTF2 and exposed to IR. Immunoblotting and IF microscopy images from these conditions are also provided (Figure S4e,f)

The major kinase responsible for phosphorylating H2AX on Ser139 and generating γ‐H2AX is ataxia‐telangiectasia mutated (ATM). As such, ATM plays a central role in DNA damage signaling and repair (Kastan & Lim, 2000). Given the large size (~350 kDa) of ATM, and the sensitivity of large cargo import to the Ran gradient (Snow et al., 2013), we posited that Ran gradient disruption in HGPS cells could reduce ATM import and help explain the defect in generating γ‐H2AX in response to IR. To test this hypothesis, we stained normal and HGPS fibroblasts for ATM, and found the latter had very low levels of nuclear ATM in both control and IR‐treated cells (Figure 6a). An antibody that recognizes phosphorylated ATM (phos‐Ser1981) also showed reduced staining in HGPS cells, though we noticed that the subset of HGPS cells with nuclear Ran also displayed p‐ATM signal (Figure 6a). By immunoblotting, ATM protein levels and p‐ATM were similar in normal and HGPS fibroblasts (Figure S3b). To test whether an ATM import defect can be induced by progerin, we first generated a GFP‐tagged form of ATM and confirmed by immunoblotting that the full‐length product GFP‐ATM can be expressed and detected in cells (Figure S4a). We then transfected GFP‐ATM alone and with HA‐Lamin A and HA‐progerin and examined the cells by IF microscopy. We found that progerin expression causes the appearance of GFP‐ATM in the cytoplasm (Figure 6b). Nuclear GFP‐ATM is observed in cells that have the import defect, indicating that either the progerin effect is partial or the GFP‐ATM is translated and imported before a progerin effect is penetrant. To address the potential concern that progerin overexpression might induce artifacts, we treated normal fibroblasts with LPV and assessed ATM and γ‐H2AX in response to IR. LPV treatment reduced the nuclear levels of ATM, p‐ATM, and γ‐H2AX (Figure S4b–d). Thus, affecting lamina structure by inhibiting the processing of endogenous lamin A alters the nuclear localization of ATM and generation of nuclear γ‐H2AX.

The data from HGPS cells and LPV‐treated normal cells suggest that chromatin regulates the generation of γ‐H2AX through the Ran protein gradient. The interphase Ran gradient could, therefore, be limiting for γ‐H2AX induction during a DNA damage response. To test this idea, we depleted the Ran import factor, NTF2, and examined the cell response to IR. Depletion of NTF2 reduced the nuclear concentration of Ran and resulted in a defect in IR‐induced γ‐H2AX generation (Figure 6c; Figure S4e,f). Plotting γ‐H2AX levels as a function of Ran N:C reveals a correlation between these values in IR‐treated cells (Figure 6d). Our data suggest that the Ran protein gradient is critical for the DNA damage response by regulating nuclear import of ATM and by affecting nuclear levels of one of its key substrates, H2AX.

## 3 DISCUSSION

A large body of literature indicates that the nuclear lamina helps organize and regulate nuclear pathways including transcription, DNA replication, nuclear size control, apoptosis, and mechanical stability (Burke & Stewart, 2013). Determining exactly how lamina proteins function in these pathways has been difficult, owing to the structural complexity and dynamic nature of the lamina (Turgay et al., 2017). One of the best‐defined functions of the nuclear lamina is its contribution to heterochromatin organization in the interphase nucleus. This involves heterochromatin contact with lamina proteins, including LAP2β, the lamin B receptor, and emerin (Gruenbaum, Margalit, Goldman, Shumaker, & Wilson, 2005). Genomewide mapping of DNA sequences that are in close proximity to the lamina has identified lamina‐associated domains (LADs), which are gene‐poor or transcriptionally silent regions constituting ~30% of nuclear DNA (Guelen et al., 2008). Thus, LADs are part of the peripheral heterochromatin that is anchored to the nuclear lamina. Physical interactions between the nuclear lamina and chromatin probably contribute to heterochromatin maintenance and provide part of the explanation for why disruption of lamina structure in HGPS causes the striking reduction in Histone H3 marks H3K9me3 and H3K27me3 (McCord et al., 2013). Loss of heterochromatin has also been shown to occur in Werner's syndrome, which is caused by mutation of a DNA helicase that is important for heterochromatin maintenance (Zhang et al., 2015). These observations suggest that heterochromatin levels could play a cellular role in premature aging (Kubben & Misteli, 2017).

The premise of our study was based on three properties of fibroblasts derived from HGPS patients, and corroboration of the properties in naïve cells: (a) compared to age‐matched controls, HGPS fibroblasts have reduced levels of heterochromatin marks H3K9me3 and H3K27me3 (Scaffidi & Misteli, 2005; Shumaker et al., 2005); (b) HGPS fibroblasts display a significant reduction in the Ran N:C protein gradient, which is positively correlated with the nuclear levels of H3K9me3, H3K27me3, and HP1α (Kelley et al., 2011), and ectopic expression of progerin in naïve cells is sufficient to reduce heterochromatin levels and disrupt the Ran gradient (Datta et al., 2014; Kelley et al., 2011); (c) treating HGPS fibroblasts with farnesyltransferase inhibitor rescues heterochromatin levels and restores the N:C Ran distribution (Kelley et al., 2011). The progerin effects on heterochromatin and Ran distribution are both dependent on a functional CAAX motif in lamin A (Snow et al., 2013), which indicates that both cellular phenotypes are initiated at the nuclear membrane. Taken together, these data suggested there could be cause and effect relationships between the structure of the nuclear lamina, the chromatin state, and the Ran system. We refer to these relationships as the Lamina–Chromatin–Ran axis.

We set out to test whether chromatin state controls the Ran gradient since this part of the axis was previously inferred from the correlation between heterochromatin levels and Ran distribution (Datta et al., 2014; Kelley et al., 2011; Snow et al., 2013). To this end, we applied inhibitors to the HMTs G9a/GLP and examined the effects on Ran distribution. Treating normal human fibroblasts with HMT inhibitors, especially Bix01294 (Kubicek et al., 2007) and UNC0638 (Vedadi et al., 2011), caused a reduction in heterochromatin and a striking disruption of the Ran gradient. The same effect was obtained by siRNA‐mediated reduction in the levels of G9a and GLP. Thus, in normal human fibroblasts, heterochromatin is critical for the interphase N:C Ran protein gradient.

Previous work has demonstrated a physical association between RCC1 and histones H2A and H2B (Nemergut, 2001), but several observations expand this view and suggest RCC1 could act as a heterochromatin‐dependent effector protein that regulates the interphase Ran gradient: (a) RCC1 is a chromatin‐associated protein that binds directly to Ran and mediates GDP‐GTP nucleotide exchange (Bischoff & Ponstingl, 1991; Ohtsubo et al., 1989); (b) the intranuclear mobility of RCC1 measured by FRAP, which reflects its chromatin dynamics, is reduced by progerin expression (Kelley et al., 2011); (c) by ChIP, RCC1 can be enriched with the heterochromatin marks on Histone 3, H3K9me3 (*p* = 0.02), and H3K27me3 (*p* = 0.02), but it does not show enrichment on chromatin marked with H3K4me3 (this study); (d) RCC1 activity is required for maintenance of the Ran gradient in interphase cells (Tachibana et al., 1994; Uchida et al., 1990); indeed, inactivation of a temperature‐sensitive allele of RCC1 results in a Ran disruption that mirrors that of progerin expression and HMT inhibition. All of these observations point to RCC1 and heterochromatin as limiting components for the Ran protein gradient. It is possible that RCC1‐independent mechanisms could influence the interphase Ran distribution, such as an interaction between heterochromatin and Ran shown to occur during mitosis (Bilbao‐Cortés, Hetzer, Längst, Becker, & Mattaj, 2002). Therefore, we also explored whether the epigenetic state of chromatin contributes to the regulation of Ran distribution in *S. cerevisiae*. We found that deletion of the methyltransferase *set2* reduced the Gsp1 N:C. This suggests that some features of the chromatin‐Ran axis are conserved between diverse species, despite the fact that budding yeast does not encode a lamin A homologue.

An intriguing feature of nucleocytoplasmic transport that emerged from our analysis of Ran defects in HGPS is that import of high molecular weight proteins is more dependent on the Ran N:C gradient than intermediate‐sized proteins (Snow et al., 2013). DNA damage response and repair components commonly exist as high molecular weight proteins, or assemble into large multi‐subunit complexes. These considerations were the basis of the prediction that the nuclear import and activity of DNA repair components might be reduced in response to Ran gradient disruption in HGPS fibroblasts. This prediction is consistent with data from multiple groups showing that HGPS cells have DNA repair defects and genomic instability, features that contribute to the biology of HGPS cells (Gonzalo & Kreienkamp, 2015; Liu et al., 2005; Liu, Wang, Ghosh, & Zhou, 2013; Manju, Muralikrishna, & Parnaik, 2006; Zhang et al., 2016; Zhang, Xiong, & Cao, 2014).

As an initial test for whether the structure of the nuclear lamina might affect DNA damage signaling through disruption of the Ran gradient, we treated normal human fibroblasts with LPV to inhibit Zmpste24‐mediated preLamin A cleavage (Coffinier et al., 2007). This approach results in disruption of the Ran protein gradient because constitutive membrane attachment of endogenous, preLamin A, like progerin, results in a loss of heterochromatin. We have shown previously that the LPV effect on the Ran gradient requires endogenous lamin A (Datta et al., 2014); thus, the phenotypes scored in our assays require preLamin A accumulation. We found that LPV treatment caused a striking reduction in the ability of normal human fibroblasts to generate nuclear γ‐H2AX in response to IR. Importantly, depleting the Ran import factor NTF2, which is a more direct approach for disrupting the Ran gradient, gave a similar defect in γ‐H2AX induction by IR. These findings indicate that the activity of the nuclear transport machinery can be limiting for an acute DNA damage response with γ‐H2AX as the readout. We found that LPV treatment reduced the nuclear level of H2AX approximately twofold, without a substantial reduction in H2AX mRNA or total protein level. We also found that LPV reduced the nuclear levels of ATM, the kinase largely responsible for H2AX phosphorylation. While it is possible that the LPV effect can be explained simply by the reduced H2AX level in the nucleus, the magnitude of the LPV effect on γ‐H2AX induction makes it seem more likely that reduction in ATM import is critical for the phenotype as well. Because the defect in H2AX nuclear localization detected by IF was partial and it was observed in only a subset of the cells, biochemical fractionation was not capable of revealing a difference in H2AX distribution (data not shown). Our conclusion that reduced nuclear levels of H2AX are explained by a transport defect is, by necessity, based strictly on single‐cell analysis. Other formal possibilities that could help explain our data from cells that have a defective nuclear lamina include selective degradation or reduced expression of nuclear H2AX in a subset of severely affected cells, or perhaps epitope masking of H2AX.

To determine whether the LPV effects on the Ran gradient are pertinent to the Ran disruption in Progeria, we exposed HGPS patient cells to IR and co‐stained for Ran and γ‐H2AX. We determined that disruption of the Ran gradient caused by endogenous progerin expression is accompanied by a significant reduction in IR‐induced γ‐H2AX generation. HGPS patient cells also displayed an ATM import defect, which was most noticeable prior to IR exposure. Each of the three HGPS lines used here (HGPS 1498, 3199, 1972) displays heterogeneity with regard to the γ‐H2AX and ATM defects, which is the case with phenotypes such as heterochromatin and progerin expression levels characterized by many groups. This underscores the importance of single‐cell measurements for the types of questions posed in this study. Because an ATM import defect could be recapitulated in normal human fibroblasts treated with LPV, we conclude that tethering of lamin A is sufficient to trigger a series of events that include altering the nuclear lamina, reduction in heterochromatin, disruption of the Ran gradient, and defective import of large protein components that are critical for marking and repairing DNA damage. The relative order of these of these events is based on (a) the Ran gradient disruption we have characterized in HGPS cells, which is reversed by FTI treatment; (b) the disruption of the Ran gradient in normal cells by LPV and by ectopic expression of progerin, including the requirement for a functional CAAX motif; (c) the effect of HMT inhibitors and G9a/GLP depletion, on the Ran gradient; (d) the observation that disruption of the Ran gradient by depleting the Ran import factor NTF2 inhibits large cargo transport and reduces γ‐H2AX induction by IR.

Our data showing that cells with a disrupted Ran gradient display a defect in IR induction of γ‐H2AX might seem contradictory to the notion that cells with altered nuclear lamina structure have a higher basal level of DNA damage and genomic instability (Gonzalo & Kreienkamp, 2015). Robust evidence that altering the nuclear lamina can generate constitutive γ‐H2AX was obtained in two different mouse models. Zmpste24^–/–^ mice were shown to have a high level of γ‐H2AX in liver (Varela et al., 2005), and G608GH/G608G mice were shown to have elevated γ‐H2AX in P5 skin fibroblasts (Osorio et al., 2011). Data indicative of constitutive γ‐H2AX generation in human HGPS cells (compared to control cells) are much less dramatic. The difference in γ‐H2AX levels in HGPS cells versus normal cells analyzed by immunoblotting was minimal (Liu et al., 2006, 2005 ). By IF microscopy, HGPS cells contain a low number of small γ‐H2AX foci (Scaffidi & Misteli, 2006). The percentage of HGPS cells with one to five foci (~90%) is larger than the percentage of fibroblasts from young donors, but overlaps old donors (Scaffidi & Misteli, 2006). Thus, in human cells a distinction can be made between the low level of basal γ‐H2AX signal detected in single cells and the large induction of γ‐H2AX that is induced by IR or chemical treatment. It also deserves mention that a low level of γ‐H2AX is generated in normal cells under routine culture conditions (McManus & Hendzel, 2005). From the available data, it appears the basal level of γ‐H2AX generated by disruption of the nuclear lamina is significantly higher in mouse models than HGPS patient cells grown in culture. Our data indicate there is a defect in DNA damage signaling involving γ‐H2AX that is based, at least in part, on nuclear transport defects. Our data are not meant to imply that disruption of the Ran gradient creates an absolute block in DNA repair, but that affected cells could have a quantitative reduction in repair events that rely on ATM and γ‐H2AX, and possibly other factors that undergo Ran‐dependent import. It can be assumed that some amount of ATM and H2AX protein partition into the nuclear compartment during nuclear envelope breakdown in mitosis, which would support damage signaling.

Disease‐associated changes in the lamina impart radiation and DNA‐damaging drug sensitivity in mice and human cell line models, but exactly how? Several groups have reported data that point to problems associated with formation of DNA repair foci, which are sometimes interpreted as kinetic effects. These include reduced recruitment of 53BP1 and Rad51 (Liu et al., 2005), and ATM (Zhang et al., 2016). Interestingly, impaired ATM activation and recruitment were proposed to explain the weak induction of γ‐H2AX in HGPS versus normal cells in response to doxorubicin (Zhang et al., 2016). Another study examining ATM function in the context of Zmpste24^–/–^ cells found a reduction in ATM activation and signaling in response to IR (Liu et al., 2013). Nuclear localization defects in the setting of HGPS cells have actually been reported for key modulators of DNA repair, 53BP1 (Cobb et al., 2016) and Parp1 (Zhang et al., 2014). In the case of Parp1, it was shown that mislocalization was correlated with Ran gradient disruption induced by progerin (Zhang et al., 2014). We propose that reduced nuclear import caused by Ran gradient disruption in Progeria contributes to the apparent change in localization of high molecular weight DNA repair factors. A global reduction in heterochromatin might, however, result in a more open structure that is permissive to DNA repair. We speculate that the more open chromatin conformation could modify the requirement for nuclear levels of repair factors like Parp1, which helps relax chromatin structure for repair reactions.

Why does reducing the nuclear level of Ran impart a bottleneck for DNA repair factors? In tracking experiments that follow the fate of NLS‐labeled quantum dots, increasing the cargo diameter from 15 to 40 nanometers caused a threefold increase in aborted attempts at nuclear import (Lowe et al., 2010). We have found that increasing the molecular mass of a cargo from 267 to 336 kDa in progerin expressing cells decreases its nuclear import (Snow et al., 2013). We also examined the transport of a DNA repair factor that exists in high molecular weight complexes, the acetyltransferase subunit Tip60, and found that it, too, has an import defect in cells expressing Progerin (Snow et al., 2013). We suggest that the strict Ran requirement for large cargo import is due to a transport step associated with targeting to, or translocation through, the NPC.

Finally, a question that emerges is whether there is a relationship between the lamina–chromatin–Ran axis and normal aging. A body of evidence indicates that epigenetic changes including a reduction in heterochromatin are associated with aging (Pal & Tyler, 2016; Zane, Sharma, & Misteli, 2014), and disrupting the Ran gradient in cancer cells can promote senescence (Cekan et al., 2016). It has also been established that the Ran gradient is susceptible to disruption by various cell stresses (Kelley & Paschal, 2007; Yasuda, Miyamoto, Saiwaki, & Yoneda, 2006). Some of the biological effects of stress signaling that occur in normal aging might, therefore, be transduced through changes in the Ran system.

## 4 MATERIALS AND METHODS

### 4.1 Cell culture

Primary human fibroblasts from HGPS patients (AGO1972, AG11498, AGO3199; designated HGPS 1972, HGPS 1498, HGPS 3199) were obtained from the Coriell Cell Repositories. Normal human fibroblasts (AGO8469; designated Normal 8469) from an unaffected father of an HGPS patient were also obtained from the Coriell Cell Repositories. Primary human fibroblasts were grown at 37°C in 5% CO_2_ in MEM (Gibco/Invitrogen, Carlsbad, CA), containing 15% FBS (HyClone), 1% MEM vitamin solution (HyClone), 1% L‐glutamine (Gibco/Invitrogen), and 1% penicillin/streptomycin (Gibco). Fibroblasts were used for experiments after being grown for ~10–19 passages. 293 T cells were obtained from ATCC. 293 T cells were grown at 37°C in 5% CO_2_ in DMEM/F12 (Gibco) supplemented with 5% FBS (Atlanta Biologicals), 1% sodium pyruvate (Gibco), 1% nonessential amino acids (Gibco), and 1% penicillin/streptomycin (Gibco/Invitrogen).

### 4.2 Immunofluorescence microscopy and image analysis of mammalian cells

Cells were grown on glass coverslips, washed with PBS, and fixed for 20 min with 3.75% formaldehyde and permeabilized in 0.2% Triton X‐100 for 5 min. Cells were blocked for 1 hr in a blocking buffer (2% FBS, 2% BSA in PBS) at room temperature. Primary antibodies were diluted in blocking buffer and incubated overnight at 4°C. Primary antibodies were used in the IF experiments: Ran mAb (BD Transduction Laboratory, #610341), H3K9me3 Rb (Abcam, #ab8898), H3K27me3 Rb (Millipore, #07–449), TPR Rb (laboratory‐prepared), ATM Rb (Novus Bio, #NB100–104), p‐ATM Rb (Abcam, #ab81292), γ‐H2AX Rb (Cell Signalling, #9718S), and γ‐H2AX mAb (Millipore, #05–636). Secondary antibodies were diluted in blocking buffer and incubated for 1 hr at room temperature. Secondary antibodies used were FITC‐labeled donkey anti‐mouse (Jackson ImmunoResearch Laboratories, Inc., #715–095–150) and Cy3‐labeled donkey anti‐rabbit (Jackson ImmunoResearch Laboratories, Inc., #711–165–152). Images shown in the figures were acquired by confocal microscopy (LSM 880; Carl Zeiss) equipped with a 40×, 1.3 NA oil immersion objective and ZEN software (Carl Zeiss). For quantification, images were acquired using an upright fluorescence microscope (Eclipse E800; Nikon) equipped with a 40×, 1.0 NA oil immersion objective, a charge‐coupled device camera (Hamamatsu Photonics; #C4742–95) and OpenLab software (PerkinElmer). All imaging was performed at ∼24°C. Nuclear and cytoplasmic signal was measured as described (Kelley & Paschal, 2007; Kelley et al., 2011) using appropriate statistical tests (Student's *t*‐test and Spearman's correlation) and calculated using Prism (GraphPad Software). Graphs were generated in GraphPad. Data were plotted as histograms, which help illustrate the ratio values within the experiment. All IF images shown were processed in parallel with Photoshop (Adobe), and figures were assembled using Illustrator (Adobe).

### 4.3 Yeast strain construction

Strains were constructed in the MATa haploid *Saccharomyces cerevisiae* strain, BY4741. GSP1, the yeast homologue of Ran, was tagged with GFP at its chromosomal locus through oligonucleotide‐directed homologous recombination with GFP‐spHIS5 amplified with primers IJM‐1 and IJM‐2 from the tagging vector, pFA6a‐link‐yoEGFP‐SpHis5 (Lee, Lim, & Thorn, 2013). Deletion of the histone methyltransferase, *set2* was performed by first amplifying the *set2* genomic locus from the set2Δ::KanMX6 strain from the Mat a haploid deletion collection (Dharmacon) with WSM‐41 and WSM‐42. This PCR product was transformed into the Gsp1‐GFP strain to delete the *set2* gene through homologous recombination. In the course of validating the deletion of *set2*, we discovered the strain contains a second copy of *set2*, which we deleted by amplifying the URA3 gene from pRSII406 (Chee & Haase, 2012) with JKM‐26 and JKM‐27. Whether the parental strain was hemidiploid for *set2* because of a gene duplication or a second copy of the chromosome is not known.

### 4.4 Yeast imaging

Yeast were imaged on an Olympus IX83 with a 60XOTIRF 1.49 NA objective, a Photometrics Prime95b camera, Xcite LED 120 Boost fluorescence light source (Excelitas), and filters for DAPI and GFP (Semrock). Cells were grown to mid‐log phase (OD600 = 0.2 to 0.6) at 30°C in Synthetic Complete Media with 2% dextrose (SCD) and then imaged on pads made of 2% agarose in SCD. Imaging was performed with an objective heater (Bioptechs) set to 30°C. GFP was imaged at 30% intensity for 200 msec with 15 z‐slices with a step size of 290 nm. For Hoechst staining, yeast were pelleted and resuspended in 2% glucose in PBS pH 7.4 with 5 µg/ml Hoechst 33342 (Molecular Probes, # H3570) followed by fixation with 3.5% paraformaldehyde for 5 min at room temperature. Cells were pelleted and then resuspended in PBS pH 7.4 and placed on an agarose pad as above. Hoechst staining was imaged as GFP was above, but with 10% excitation intensity. Images were deconvolved using Huygens (SVI) with the CMLE algorithm and a signal to noise ratio of 4. Images were quantified using FIJI.

### 4.5 Drugs, radiation, and siRNA

The HMT inhibitors used were Bix01294 (Sigma‐Aldrich, #B9311), UNC0638 (Sigma‐Aldrich, #U4885), and A‐366 (Sigma‐Aldrich, #SML4110). The HMT inhibitors were dissolved in DMSO and tested in the 1–10 micromolar range to identify concentrations that reduce Histone H3K9me3 levels with minimal toxicity (BIX01294**,** 2 µM, 24 hr; UNC0638, 3 µM, 24 hr; A‐366, 3 µM, 72 hr). The Zmpste24 protease inhibitor lopinavir (LPV; Cayman Chemicals, #13854) was dissolved in DMSO and used as described (20 µM, 72 hr) to inhibit proteolytic processing of prelamin A. Human fibroblasts were exposed to ionizing radiation (5 Gy), returned to the incubator for 30 min, and subsequently analyzed by IF microscopy. The siRNA to the Ran import factor NTF2 (Santa Cruz, #sc‐36105) and a control siRNA (Fisher, #AM4635) were introduced into normal human fibroblasts (80% confluence) at a concentration of 10 µM using Lipofectamine RNAiMAX (Invitrogen). The cells were analyzed ~96 hr post‐transfection.

### 4.6 Chromatin immunoprecipitation

293 T cells were transfected with pK‐RCC1‐Flag and empty vector using FuGENE 6 (Promega) according to the manufacturer's protocol and after 24 hr used for ChIP. Cells were formaldehyde cross‐linked (1%, 15 min), quenched with glycine (125 mM, 5 min), harvested, and resuspended in lysis buffer (1% SDS, 10 mM EDTA, 50 mM Tris pH 8.0, 1 mM PMSF, 1 µg/ml each of aprotinin, leupeptin, and pepstatin. The sample was disrupted (Branson Sonifier 250) on ice using a tip sonicator (0.7 s on, 1.3 s off, 40% power) to obtain DNA fragments in the 200–500 bp range. After centrifugation, the supernatant was combined with five volumes of ChIP dilution buffer (1.1% Triton X‐100, 1.2 mM EDTA, 16.7 mM Tris pH 8.0, 167 mM NaCl, 1 mM PMSF, 1 µg/ml each of aprotinin, leupeptin, and pepstatin). Immunoprecipitation was performed with anti‐FLAG antibody (5 µg; M2 affinity gel, Sigma‐Aldrich, #A2220) rotating overnight at 4°C. After a series of washes, the cross‐linking was reversed, and beads were eluted with gel sample buffer and analyzed by immunoblotting with antibodies to RCC1, Histone H3K9me3, H3K27me3, and H3K4me3. Immunoblots were incubated with fluorescently labeled secondary antibodies, and scanned and detected on the Odyssey Infrared Imager (LICOR). The experiment was performed three times, the enrichment of each mark was quantified, and the data pooled and displayed as a histogram.

## CONFLICT OF INTEREST

None declared.
