# Supplementary figures and images for: A nuclear lamina‐chromatin‐Ran GTPase axis modulates nuclear import and DNA damage signaling
Source: Aging Cell. 2018 Dec 19;18(1):e12851. doi: 10.1111/acel.12851 (PMC6351833; doi:10.1111/acel.12851)

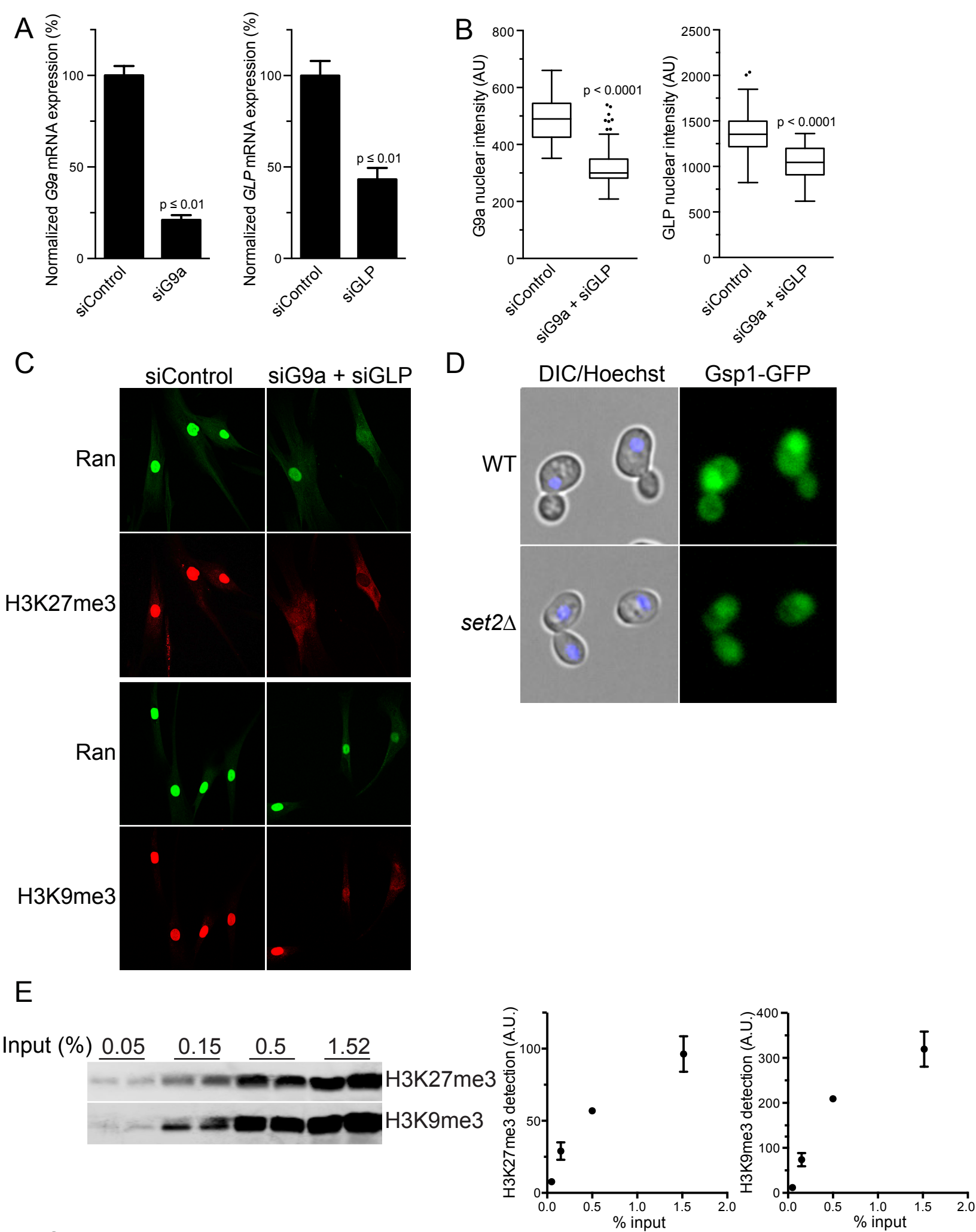

Fig.S1

Supplement: Supplementary file 1 [file ACEL-18-e12851-s001.pdf]

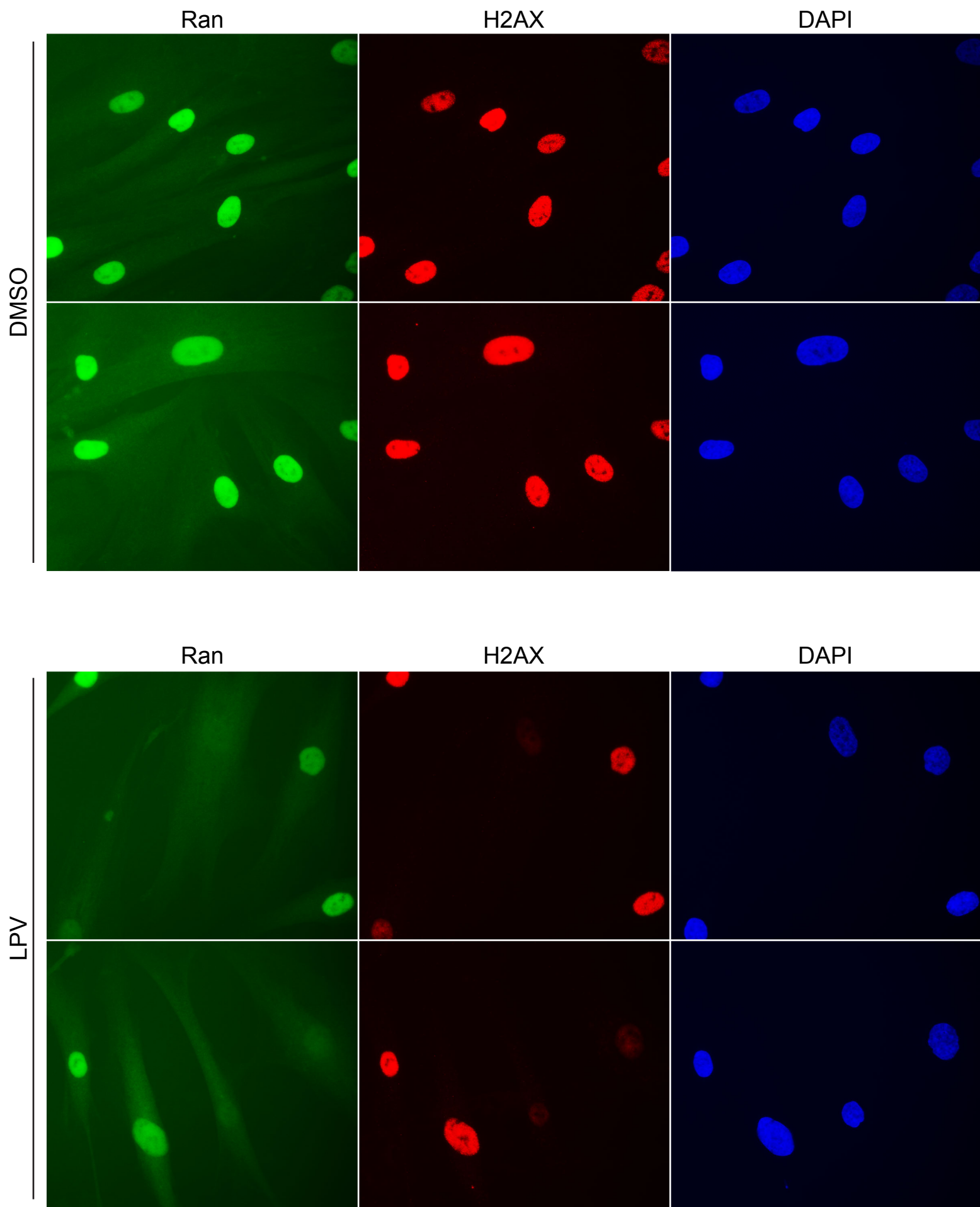

Fig. S2

Supplement: Supplementary file 2 [file ACEL-18-e12851-s002.pdf]

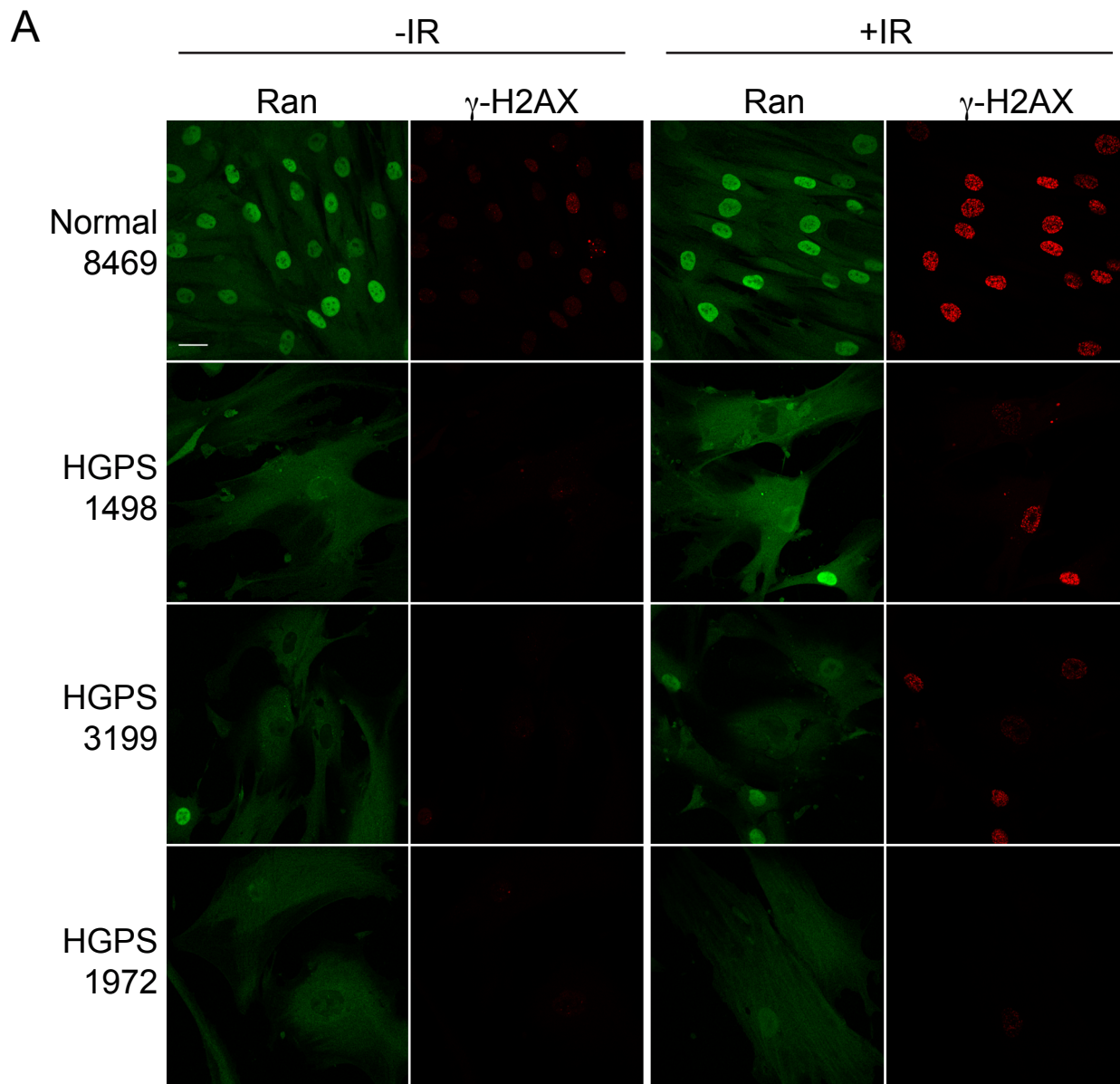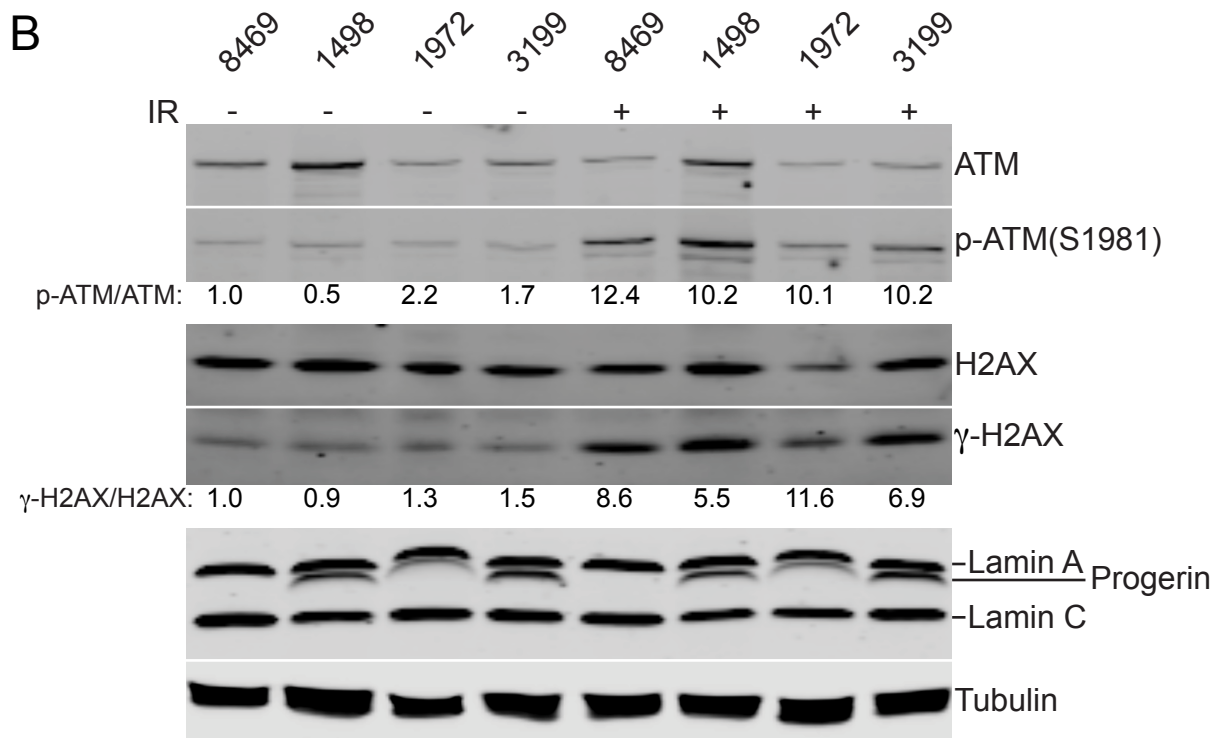

Fig. S3

Supplement: Supplementary file 3 [file ACEL-18-e12851-s003.pdf]

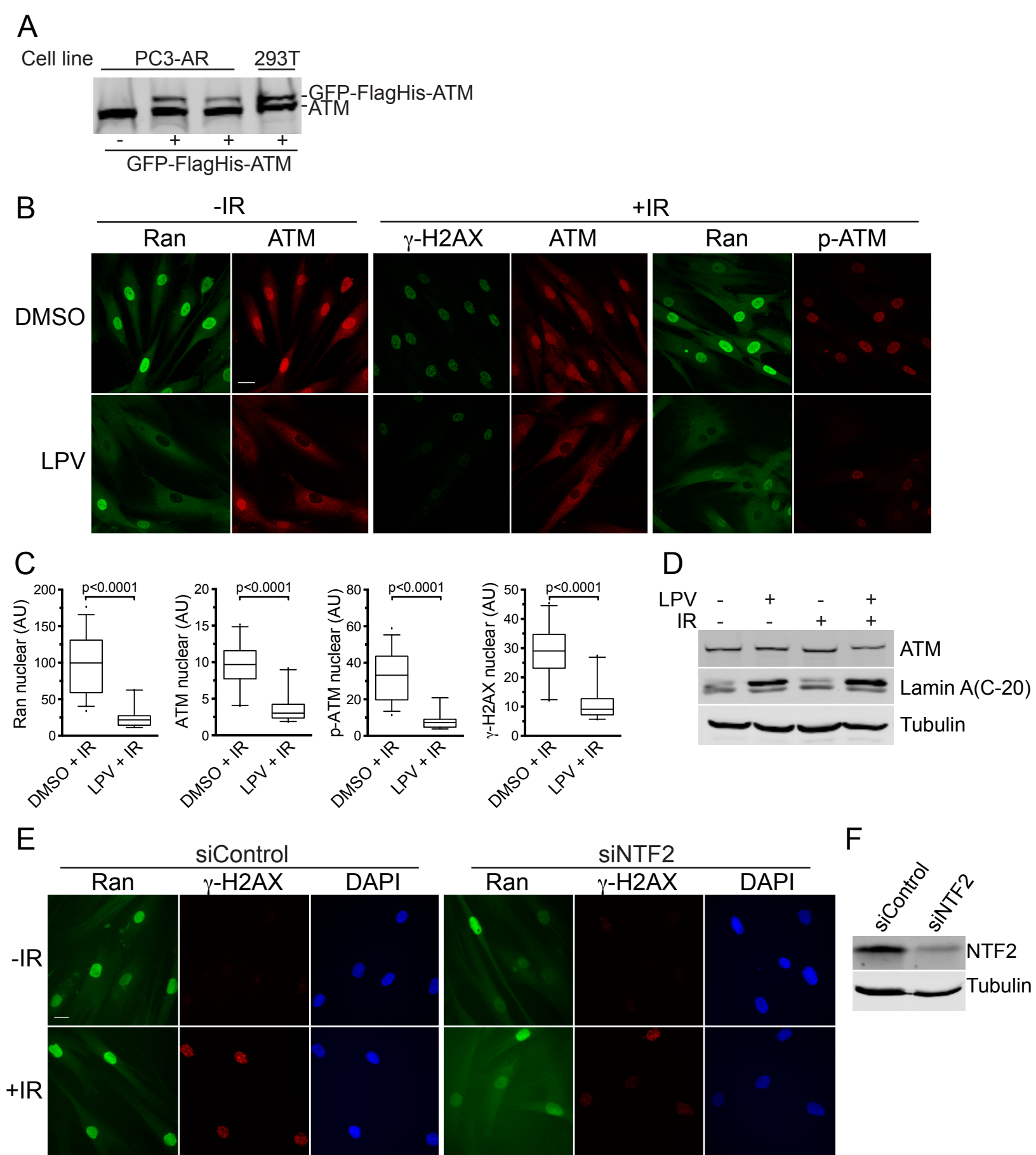

Fig. S4

Supplement: Supplementary file 4 [file ACEL-18-e12851-s004.pdf]
